# Application of functional near-infrared spectroscopy in evaluating antidepressant treatment efficacy: a review of recent evidence (2021–2026)

**DOI:** 10.3389/fpsyg.2026.1934277

**Published:** 2026-09-07

**Authors:** Mao-liang Ma, Jiang Cao, Jian-li Yang

**Affiliations:** 1 Department of Clinical Psychology, Tianjin Medical University General Hospital Airport Hospital, Tianjin, China; 2 Department of Clinical Psychology, Tianjin Medical University General Hospital, Tianjin, China

**Keywords:** biomarker, brain functional imaging, functional near-infrared spectroscopy, major depressive disorder, treatment outcome evaluation

## Abstract

Major depressive disorder (MDD) remains challenging to treat due to the lack of objective biomarkers for monitoring treatment response and predicting outcomes. Traditional treatment response evaluation rely heavily on subjective assessments, creating a pressing need for objective biomarkers to monitor therapeutic efficacy. Functional near-infrared spectroscopy (fNIRS), a non-invasive, portable, and high-temporal-resolution brain imaging technique, can indirectly reflect brain function by monitoring metrics such as oxy-hemoglobin. This review systematically synthesizes research from 2021–2026 to detail the application of fNIRS in evaluating various depression interventions, including pharmacotherapy, physical therapies, psychological interventions, integrated interventions, and combined East–West medicine approaches. Evidence indicates that fNIRS can precisely capture hemodynamic and functional connectivity changes in key brain regions like the prefrontal and temporal cortices across different treatments, which correlate closely with clinical symptom improvement. This positions fNIRS as a promising biomarker for treatment efficacy assessment and a potential tool for predicting therapeutic response. Current limitations, such as small sample sizes and a lack of standardized protocols, are also discussed. Future directions include promoting multimodal integration, establishing standardized procedures, and building machine learning models to advance personalized and precise treatment monitoring for depression.

## 1 Introduction

MDD affects approximately 2–5 percent of the global population, with over 300 million people diagnosed. It ranks as the leading cause of disability worldwide, and this prevalence is rising (Ferrari et al., 2013; Berton and Nestler, 2006). Depressive symptoms can severely disrupt daily activities, leading to substantial social and economic burdens (Proudman et al., 2021). The etiology of depression remains multifactorial, encompassing life stressors, chronic illness (such as hypertension and diabetes), pain, medication reactions, and genetic factors (Barnett, 2019). Early identification and proper management of depression are essential for enhancing patient outcomes. Traditional diagnostic and monitoring approaches mainly rely on patient self-reported symptoms and clinical scale evaluation, which tend to be relatively subjective and exhibit significant variability between individuals (Yasin et al., 2021). Therefore, identifying objective biomarkers to monitor treatment efficacy and guide clinical decision-making holds significant importance for depression diagnosis and management. Biomarkers, serving as objective indicators reflecting biological processes or conditions, provide important insights into individual susceptibility to depression and potential responses to specific treatment modalities. They play a crucial role in improving and expediting therapeutic outcomes (Poirot et al., 2024). Among various potential biomarkers, neuroimaging techniques are particularly notable for directly examine brain function and structure (Stangl et al., 2023). An increasing volume of research employing neuroimaging techniques has made notable progress in predicting treatment responses to psychiatric disorders, such as depression, while also shedding light on the underlying pathophysiological mechanisms (Phillips et al., 2015). fNIRS is an emerging non-invasive brain imaging technique that uses infrared light at wavelengths ranging from 650 to 900 nanometers to illuminate the head. It measures relative concentration changes of oxygenated hemoglobin (HbO), deoxygenated hemoglobin (HbR), and total hemoglobin (HbT) by utilizing the absorption of near-infrared light by hemoglobin. Based on neurovascular coupling mechanisms, it provides an indirect reflection of cerebral metabolic regulation. fNIRS offers moderate spatial resolution and penetration depth, coupled with high temporal resolution. Its benefits include non-invasiveness, safety, portability, and the ability to monitor in real time (Hou et al., 2021). It is commonly employed alongside cognitive or motor tasks to activate targeted cortical regions of interest without interfering with therapeutic effects. It is now extensively applied in clinical research for a range of conditions, including depression, bipolar disorder, schizophrenia, and autism spectrum disorders (Agbangla et al., 2017; Tassi et al., 2022). Studies using fNIRS measurement have demonstrated correlations between frontal hemodynamic responses and the severity of depression (Nishizawa et al., 2019). Recent large-scale cohort studies have also revealed that neurological disorders such as epilepsy are associated with significantly elevated risk of subsequent depression, particularly in the early post-diagnostic period. This highlights the urgent need for objective, neuroimaging-based biomarkers like fNIRS for treatment monitoring in comorbid populations (Bae et al., 2025b; Bae et al., 2025a).

Against this backdrop, this paper reviews recent developments in the use of fNIRS for assessing and monitoring the therapeutic efficacy of depression treatments between 2021 and 2026. Specifically, we examine fNIRS applications across five major treatment modalities: pharmacological interventions, physical therapies, psychotherapy, integrated interventions, and combined traditional Chinese and Western medicine. This review is organized by therapeutic modality for three reasons. First, different interventions engage distinct neural mechanisms, which poses challenges for directly comparing their fNIRS correlates across modalities. Second, clinicians typically select a treatment modality first and then seek prognostic biomarkers that are informative for the chosen modality, which may make a modality-based structure more clinically actionable in practice. Third, the existing literature itself is clustered by intervention type, with few studies directly comparing modalities, which complicates a cross-modality synthesis at this stage; however, indirect comparisons may still offer preliminary insights. Rather than addressing diagnostic applications—which have been reviewed extensively elsewhere (Tong et al., 2024; Karna and Verma, 2026)—this review is deliberately scoped to focus on treatment evaluation, which is considered one of the most promising clinical applications of fNIRS. Within each modality, we organize findings into 1–3 functional dimensions: (1) monitoring treatment-induced hemodynamic changes, (2) elucidating underlying neural mechanisms, and (3) predicting individual therapeutic outcomes. Through this structured synthesis, we aim to advance understanding of the neurobiological basis of treatment response in depression and provide a practical reference for researchers and clinicians using fNIRS for treatment monitoring.

## 2 Application of fNIRS in antidepressant pharmacotherapy

Numerous studies have demonstrated that neuroimaging techniques aid in the classification of depressive disorders and the prediction of treatment outcomes (Chen et al., 2021). However, current neuroimaging research on first-episode medication-naïve depressive disorder (FMD) predominantly focuses on cross-sectional designs utilizing resting-state fMRI. There is a lack of longitudinal studies employing task-based neuroimaging techniques, which prevents elucidating neuroimaging alterations during the progression of depression (Helwig and Snodgress, 2019).

### 2.1 Monitoring treatment efficacy

fNIRS enables objective and dynamic monitoring of antidepressant efficacy by tracking longitudinal changes in hemodynamic signals. Wang H. et al. (2025) employed 52-channel fNIRS technology to compare activation levels in the frontal-temporal regions between FMD patients and healthy controls (HCs) during the Verbal Fluency Task (VFT), examined changes in brain activation before and after treatment in FMD patients, and explored the relationship between brain activation and clinical presentation. Patient medication was expressed in equivalent doses of imipramine. The results showed FMD patients exhibited significantly reduced activation in multiple brain regions (including DLPFC, Broca’s area, and orbitofrontal cortex) compared to HCs at baseline, 4 weeks, and 8 weeks of treatment; at 8 weeks, they also showed reduced activation in additional regions and increased activation in the middle temporal gyrus, suggesting specific brain regions may aid diagnosis and reflect treatment efficacy. Integrating Hamilton Depression Scale (HAMD) findings with fNIRS reports reveals inconsistent activation changes across frontal-temporal regions in FMD patients despite marked symptom improvement. This not only reflects the antidepressant effects on fNIRS signals but also implies a disconnect between clinical symptoms and brain activity.

Research indicates alterations in HbO and HbR levels among depressed patients, primarily occurring in the prefrontal cortex associated with emotional regulation, decision-making, and cognitive control (Sinko et al., 2022). Huang and Shan (2025) focused on the prefrontal cortex and conducted a longitudinal study to monitor changes in HbO and HbR levels during treatment with a selective serotonin reuptake inhibitor (SSRI). The report indicated that alongside clinical symptom improvement, the MDD group exhibited elevated HbO and reduced HbR post-treatment, with both levels remaining significantly different from HCs at 4 weeks. By 24 weeks, HbO increased further while HbR decreased further, with no statistically significant difference from HCs. This indicates that after 24 weeks of SSRI treatment, patients with depression exhibit enhanced cerebral oxygenation and significant improvements in cerebral hemodynamics and neurovascular function (Zhang F. et al., 2023). It also highlighted fNIRS’ value in early diagnosis and treatment monitoring.

### 2.2 Describing neural mechanisms

fNIRS explores the neural mechanisms underlying antidepressant pharmacotherapy by detecting hemodynamic changes in the prefrontal and temporal cortices. Within the observational research paradigm targeting specific depressive symptoms, Chen L.-F. et al. (2025) focused on anhedonic depression, applying fNIRS to assess cerebral blood flow in patients treated with agomelatine (25 mg/day). They found reduced activation in multiple brain regions at 1, 4, and 8 weeks, improved global and local efficiency via graph-theoretic network analysis at week 1, and a positive correlation between left OFC HbO levels and reduced Montgomery-Asberg Depression Rating Scale (MADRS) scores. Previous studies have linked the OFC to punishment and reward responses (Gourley et al., 2016; Kringelbach and Rolls, 2004), and recent multimodal findings indicate a negative correlation between pretreatment anhedonia symptom scores and medial structural connectivity of the OFC (Donnelly et al., 2024). Together, these findings suggest that abnormal functional connectivity in the OFC may reflect the pathophysiological process of anhedonic depression and could guide early treatment.

### 2.3 Predicting treatment response

Recent studies have explored the value of fNIRS in predicting antidepressant response, especially using machine learning models. In a 6-month longitudinal study involving 70 patients with MDD. Ho et al. (2025) employed machine learning algorithms to evaluate the predictive capability of fNIRS and clinical test data for response to medication (fluoxetine equivalent dose). They tested whether fNIRS single-modality classification outperformed dual-modality classification combining fNIRS and clinical information. Experimenters observed significant correlations between HAMD score reduction and task-related 52-channel HbT changes. After assigning the 52 channels to five brain regions, significant differences in mean HbT values or task-related changes were also found between treatment responders and non-responders. Analysis within machine learning models demonstrated that fNIRS features derived solely from task-related HbT changes across multiple brain regions yielded comparable or superior predictive performance for MDD treatment response compared to either clinical measurements alone or a combination of clinical measurements and fNIRS features. Notably, although the study used nested cross-validation with LOOCV as the external test loop and stratified five-fold cross-validation as the internal validation loop to reduce overfitting, independent validation using a completely separate patient cohort would be necessary to further strengthen the generalizability and clinical utility of these predictive models.

### 2.4 Summary of fNIRS research on antidepressant pharmacotherapy

A brief overview of fNIRS studies in antidepressant pharmacotherapy is presented in (Table 1). In summary, MDD patients consistently show reduced prefrontal and temporal activation relative to healthy controls, along with abnormal HbO/HbR levels. Pharmacological mechanisms differ: SSRIs gradually normalize prefrontal hemodynamics and neurovascular coupling, whereas agomelatine primarily repairs orbitofrontal functional connectivity. However, whether hemodynamic changes correlate with clinical improvement remains inconsistent across studies—likely due to variations in cognitive tasks, treatment duration, or medication type. Regarding prediction, machine learning applied to task-related fNIRS features (e.g., HbT changes) shows promise, but independent validation in larger cohorts is still lacking. The four pharmacological studies reviewed in this section had a median sample size of 72 (range 60–84), with all studies exceeding 30 participants, suggesting relatively satisfactory statistical robustness. However, considerable heterogeneity in cognitive tasks, drug types, and treatment durations across studies limits direct comparisons.

**Table 1:** A brief summary of fNIRS in antidepressant pharmacotherapy.

Author	Sample	Treatment type	fNIRS instrument	Task paradigm	Brain region	Measurement index	Main conclusions
Wang H. et al. (2025)	30FMD, 30HCs	Equivalent doses of imipramine.	52-channel, ETG-4100, Hitachi	VFT, letter fluency tasks (LFTS) and category fluency tasks (CFTs).	Frontal and temporal cortex	HbO, HbR, and HbT	The superior temporal gyrus and inferior central gyrus may serve as sensitive neuroimaging markers for evaluating the clinical efficacy of MDD
Huang et al. (2025)	40FMD, 40HCs	SSRI	33-Channel	cognitive tasks	Prefrontal cortex	HbO and HbR levels	fNIRS effectively detects cerebral hemodynamic changes in MDD, with post-SSRI improvements in HbO and HbR paralleling clinical outcomes
Ho et al. (2025)	70 MDD patients	Fluoxetine equivalent dose	52-channel, ETG-4000, Hitachi	VFT	pSFC, dlPFC, STG, vlPFC, mPFC	HbO, HbR, and HbT	Task-related HbT changes significantly distinguished MDD treatment responders from non-responders and, as machine learning features, outperformed clinical measurements in predicting treatment outcomes.
Chen L.-F. et al. (2025)	84 patients with anhedonic depression	Agomelatine (25 mg/day)	48-channel, NIRSIT	VFT and a 2-back task	DLPFC, VLPFC, FPC, OFC	HbO	Abnormal OFC functional connectivity may reflect anhedonia’s pathophysiology and serve as an early treatment-guiding biomarker.

## 3 Application of fNIRS in physical therapy for depression

### 3.1 Application of fNIRS in transcranial magnetic stimulation for depression

The US Food and Drug Administration (FDA) has approved the use of repetitive transcranial magnetic stimulation (rTMS) and intermittent theta burst stimulation (iTBS) for depression unresponsive to standard pharmacological treatments. Substantial evidence supports the efficacy of high-frequency rTMS or iTBS, particularly when targeting the left dorsolateral prefrontal cortex (DLPFC), yielding significant antidepressant effects (Chou et al., 2021). However, treatment response rates and patient variability remain considerable (Kaster et al., 2019). Cole et al. (2020) applied functional magnetic resonance imaging (fMRI) to identify specific neural circuits linking the DLPFC and the rostral anterior cingulate cortex (rACC), exhibiting the neurobiological significance of this circuitry in rTMS treatment. Compared to fMRI, fNIRS offers advantages of high ecological validity, superior temporal resolution, and reduced susceptibility to electromagnetic interference, thereby playing an equally significant role in elucidating neurophysiological changes associated with rTMS.

#### 3.1.1 Neural mechanism and efficacy monitoring

In recent years, Japanese researchers have published several studies utilizing fNIRS to evaluate rTMS treatment for depression. Kawabata et al. (2022) employed fNIRS to measure cerebral blood flow in 15 patients with moderate depression before and after 30 sessions of high-frequency rTMS targeting the left DLPFC. in order to investigate whether rTMS treatment can improve the prefrontal cortical dysfunction in depressed patients. The analysis revealed a significant reduction in patients’ total HAMD scores post-treatment, accompanied by a marked increase in the mean HbO integral values (reflecting cerebral activation) in the frontal and temporal cortices, with values approximately 10 times higher in the post-treatment phase compared to pre-treatment. However, the small sample size (*n* = 15) limits generalizability of these findings.

Similar findings were reported in studies by Chou et al. (2023), who pioneered the use of 52-channel fNIRS to investigate the relationship between frontal lobe activity and symptom changes following rTMS treatment, while also comparing frontal lobe activity during the VFT between responders and non-responders to rTMS therapy. Results revealed that, regardless of whether HAMD scores decreased significantly, all patients showed increased frontal lobe activity after 20 sessions, but responders exhibited a more sustained and significant increase, with this increase correlating significantly with symptom improvement at 10 and 20 sessions— a correlation not observed in non-responders, suggesting fNIRS-monitored frontal activity could serve as a biomarker for rTMS efficacy.

Chinese researchers Huang et al. (2022) adopted fNIRS to investigate the effects of low-frequency rTMS (2 Hz) to the right DLPFC on prefrontal activity in depressed patients. After four weeks of treatment, HbO levels increased in the bilateral frontal polar prefrontal cortex (FPPFC), ventrolateral prefrontal cortex (VLPFC), and left DLPFC during VFT; the post-treatment elevation in right VLPFC HbO levels positively correlated with HAMD score reduction, indicating 2 Hz rTMS can enhance prefrontal function in depressed patients.

In the field of depression comorbid with somatic diseases, Zhao et al. (2025) utilized fNIRS technology to explore the effects of bilateral rTMS treatment (1 Hz right DLPFC and 10 Hz left DLPFC) on cortical activation in patients with Alzheimer’s disease (AD) comorbid with depression. Results revealed a decrease in HBO concentration in right DLPFC-related leads from baseline to week 4. Improvement in HAMD scores was negatively correlated with reduced hemodynamic response in this brain area. These observations support a potential mechanism for rTMS-mediated improvement in depression among AD patients: namely, reduced metabolic activity and blood flow perfusion in specific regions of the right DLPFC.

Intermittent theta burst stimulation (iTBS) is a relatively novel form of rTMS, applied for significantly shorter durations yet demonstrating comparable clinical efficacy to standard protocols (Blumberger et al., 2018). Struckmann et al. (2021) applied a 2-channel fNIRS device to measure oxyhemoglobin responses in the dmPFC during iTBS treatment in patients with depression, finding iTBS modulated local prefrontal oxyhemoglobin responses, with increased levels observed one and 2 weeks post-intervention. However, these measurements were limited by low spatial resolution and coverage, failing to elucidate underlying brain network alterations. Complementing the longitudinal observation, in a cross-sectional design (Jin et al., 2026), a single standardized brain stimulation session served as an exogenous perturbation, with fNIRS continuously recording prefrontal hemodynamic signals before, during, and after iTBS to investigate state-dependent differences in brain reactivity to identical stimulation. During iTBS, HbR in the left DLPFC increased across all participants, yet the elevation was significantly larger in both the current and remitted depression groups compared with healthy controls. Following stimulation, the current depression group exhibited a significantly greater HbO increase than controls. In addition, the number of prior depressive episodes emerged as a significant independent predictor of post-stimulation HbO augmentation, suggesting that progressive impairment of prefrontal neuroplasticity may underlie reduced treatment responsiveness in chronic or recurrent depression.

Beyond AD, iTBS also shows promise in RCTs for Parkinson’s disease with comorbid depression (PD-D). Hou et al. (2026) first demonstrated the clinical efficacy of iTBS for PD-D and explored its neural mechanisms using fNIRS cerebral oxygenation and dynamic network metrics. fNIRS data revealed that, in parallel with marked symptomatic improvement, both iTBS and rTMS induced significantly larger increases in left DLPFC activation relative to sham, along with significantly enhanced left frontal pole activation. Dynamic functional connectivity analyses further demonstrated that both active treatments significantly reduced the dwell time in low-connectivity states, offering network-level evidence for their mechanisms. Of note, iTBS also confers a time-saving advantage.

Unlike most of the studies where subjects maintained prior medication regimens, Zhang and colleagues (Zhang Y. et al., 2023) adopted fNIRS to assess the efficacy of iTBS alone as an intervention for anxiety and depression. Post-treatment, patients showed significantly enhanced hemodynamic activity in Broca’s area and frontal regions; functional connectivity between all brain regions strengthened, with significantly higher intensity between regions of interest (ROIs) post-intervention, further confirming iTBS’s positive role in alleviating symptoms and enhancing brain connectivity.

#### 3.1.2 Describing neural mechanisms

Building on the team’s previous findings on neural mechanisms and immediate efficacy monitoring, Struckmann et al. subsequently conducted another study (Struckmann et al., 2022) combined fNIRS and fMRI to explore the neural network mechanisms underlying iTBS for depression, with an exploratory analysis of whether fNIRS signals could predict subsequent neural changes. fNIRS was used to measure oxyhemoglobin changes in the left and right DLPFC during dmPFC-iTBS, and these regions were used as seed regions for resting-state fMRI functional connectivity analysis. Symptomatic improvement was correlated with reduced connectivity between the DLPFC and insula, while increased fNIRS oxyhemoglobin levels were associated with diminished connectivity to the posterior parietal cortex (PPC). The insula is a key node of salience network (SN) and is highly connected with other brain regions, DLPFC and PPC are the main nodes of the central executive network (CEN) (Seeley et al., 2007). Importantly, fNIRS signals acquired on the first day of treatment predicted subsequent neuroplastic changes in functional connectivity rather than final clinical treatment response. It should be noted that fNIRS and fMRI data were not collected simultaneously in this study.

Beyond characterizing neural mechanisms and monitoring real-time efficacy, fNIRS also shows unique value in predicting individual treatment responses to rTMS and iTBS. Tsuji et al. (2025) adopted a personalized approach, using fNIRS technology to evaluate frontal lobe hemodynamic changes based on HAMD-classified disease severity. Patients in the mild and severe groups exhibited significantly greater frontal pole hemodynamic activity than those in the moderate group. Within the frontal pole, the HAMD improvement was significantly greater in the group with increased integral values than in the group with decreased integral values. That is, patients with heightened frontal lobe activation also demonstrated marked improvement in depressive symptoms, suggesting a potential correlation between rTMS-induced frontal hemodynamic changes and enhanced treatment outcomes. The authors hypothesized that the enhanced rTMS-induced cerebral hemodynamic response may be associated with heightened neuroplasticity and normalized neurotransmitter balance, potentially reactivating impaired brain functions.

The laterality index (LI), calculated from task-related frontal oxygenation responses, may correlate with rTMS treatment efficacy (Tamashiro et al., 2019), Yamazaki et al. (2022) enrolled 19 depressed patients undergoing high-frequency rTMS. First, changes in LI before and after rTMS treatment were compared. Second, reductions in MADRS scores were compared between the right-dominant group (pre-LI < 0) and the left-dominant group (pre-LI ≥ 0). Results showed significant post-treatment alterations in LI for VFT-task-related oxygenation responses. The right-dominant group exhibited greater MADRS score reductions than the left-dominant group, the lateralization of task-related hemodynamic responses in the prefrontal cortex shifted leftward, suggesting that the pre-LI calculated using fNIRS data could be a potential predictor of rTMS efficacy in patients with depression. Given the modest sample size (*n* = 19), these findings require replication in larger cohorts.

In addition to the LI proposed by Yamazaki et al., a recent study (Lin et al., 2025) has employed fNIRS as a biomarker for treatment-resistant depression (TRD). They found that baseline hypo-oxygenation in the OFC is a risk factor for non-response to iTBS, lower task-related HbO activation in the left DLPFC was associated with poor subsequent clinical improvement, fNIRS-derived prefrontal hemodynamic responses were significantly associated with changes in MADRS scores, these results validate the feasibility of fNIRS as a stratification tool for TMS efficacy in treatment-resistant depression.

#### 3.1.3 Summary of fNIRS research on transcranial magnetic stimulation

A brief overview of fNIRS studies in transcranial magnetic stimulation for depression is presented in (Table 2). In summary, MDD patients exhibit reduced prefrontal hemodynamic responsiveness and abnormal lateralization. Unlike antidepressants, which indirectly modulate brain function, rTMS generally increases prefrontal activation and neural plasticity, and iTBS can also modulate local hemodynamics and network connectivity. Increased HbO integral values and strengthened connectivity are consistently associated with clinical response. The laterality index (LI) derived from fNIRS may serve as a predictor of rTMS outcome. Recent evidence further extends these findings: baseline hypo-oxygenation in the OFC and blunted acute hemodynamic responses to iTBS predict poor outcomes in treatment-resistant and recurrent depression, respectively, while iTBS also shows efficacy in PD-D by normalizing left DLPFC and FPA activation. Notably, not all studies report uniform activation increases; some find region-specific decreases, highlighting the need for standardized protocols. Other studies has also pointed out that patients with MDD exhibit blunted frontostriatal coupling during reward tasks and reduced DLPFC activation during tasks requiring emotion regulation. In contrast, activation of the ACC and amygdala is enhanced in response to emotional cues, and frontostriatal coupling is increased during negative processing. It is therefore speculated that the direction of frontal activation in depressed patients depends on task state (Pizzagalli and Roberts, 2022). It should also be noted that rTMS can induce systemic cardiovascular changes (e.g., heart rate, blood pressure, autonomic arousal), and the observed fNIRS signal alterations may partly reflect such physiological confounds rather than pure cortical activation. Therefore, HbO changes should be interpreted as indirect markers of neural modulation, especially when drawing conclusions about localized prefrontal excitability. Notably, the 12 TMS studies reviewed showed considerable variation in sample size (median 40.5, range 15–121), with three studies (25%) having fewer than 30 participants. These small-sample studies limit the statistical robustness and generalizability of the findings, underscoring the need for larger confirmatory cohorts.

**Table 2:** A brief summary of fNIRS in transcranial magnetic stimulation for depression.

Author	Sample	Treatment type	fNIRS instrument	Task paradigm	Brain region	Measurement index	Main conclusions
Kawabata et al. (2022)	15 patients with moderate depression	High-frequency rTMS	15-channel f OEG-17ME	VFT	The frontal and temporal cortices	HbO integral values	Thirty sessions of rTMS elicited a notable increase in oxygenated hemoglobin, reflected as widespread elevation in blood flow across multiple frontal cortex channels.
Chou et al. (2023)	26 MDD patients	10 Hz rTMS	52-channel, ETG-4000; Hitachi	VFT	The bilateral frontal region (R1)	HbO	Responders and non-responders to rTMS exhibited distinct frontal lobe activity patterns, underscoring fNIRS’s promise as a biomarker for monitoring treatment response in MDD.
Tsuji et al. (2025)	45 patients with depression	10 Hz rTMS	15-channel OEG-17ME, Spectratech	VFT	FPA, DLPFC, VLPFC, L-LFR, R-LFR	Integral values which were calculated from the HbO and HbR	Patients showing greater frontal lobe activation exhibited more pronounced symptom improvement, indicating that rTMS-induced hemodynamic changes may contribute to better clinical outcomes.
Yamazaki et al. (2022)	19 MDD patients	10 Hz rTMS	16-channel Spectratech OEG-16	VFT	left PFC	HbO, the laterality index (LI) based on the task-related oxygenation response	The pre-LI derived from fNIRS data holds promise as a predictor of rTMS efficacy in patients with depression.
Lin et al. (2025)	100 TRD	iTBS, active stimulation (*n* = 50), sham stimulation (*n* = 50)	48-channel, NIRSIT; OBELAB, Korea	resting-state, VFT, Two-back task	DLPFC, VLPFC, FPC, OFC	HbO. HbR	Task-induced prefrontal hemodynamic responses, particularly in the left DLPFC and OFC, may function as predictive biomarkers of antidepressant efficacy in TRD.
Huang et al. (2022)	40FMD, 40HCs	2 Hz rTMS	37 multi-channel, BS-3000	VFT	DLPFC, VLPFC, the frontopolar PFC	HbO	Prefrontal activation during cognitive tasks, as measured by fNIRS, holds promise as a biomarker for monitoring rTMS treatment response.
Zhao et al. (2025)	33 patients with AD and depression	Bilateral rTMS (1 Hz rTMS and 10-Hz rTMS)	48-channel NirScan, Danyang Huichuang	VFT	Bilateral PFC and temporal cortices	HbO	Prefrontal activation measured by fNIRS may serve as a biomarker for monitoring rTMS treatment response in depressed patients.
Struckmann et al. (2021)	39TRD	iTBS	2-channel NIRO-200NX	Resting state	Corticolimbic network	HbO	iTBS induced a sustained increase in local prefrontal oxyhemoglobin levels, evident at both one and two weeks post-treatment.
Struckmann et al. (2022)	42 patients with ongoing depression	iTBS	2-channel CW-NIRS, NIRO 200X	Resting state	DLPFC	The network effects behind fNIRS response	fNIRS responses to iTBS were modulated by changes within both the salience network and the central executive network
Jin et al. (2026)	39 current MDD, 41 remission MDD, and 41HCs	iTBS	28-channel NIRx, Germany	Concurrent rTMS/fNIRS paradigm	the bilateral PFC	HbO, HbR, FC, global network measures	Post-iTBS prefrontal hemodynamic response amplitude significantly correlated with current depression severity and past episode count.
Zhang Y. et al. (2023)	37 patients with anxiety and depression	iTBS	53-channels	VFT	Prefrontal cortex	HbO	fNIRS detected post-iTBS increases in regional blood oxygen and cortical activation, coupled with enhanced interregional connectivity.
Hou et al. (2026)	54PD-D	iTBS, rTMS	53-channel BS-7000 system	VFT	DLPFC, FPA, FEF	HbO, brain connectivity	iTBS can serve as an efficient and practical therapy for PD-D

### 3.2 Application of fNIRS in transcranial electrical stimulation for depression treatment

The latest evidence-based medical guidelines recommend that transcranial direct current stimulation (tDCS) with anodal stimulation of the left DLPFC is effective in treating neurological and psychiatric disorders (Campos et al., 2024), However, the antidepressant efficacy of tDCS remains partially contested (Borrione et al., 2024). Further investigation into the mechanisms and neurophysiology underlying tDCS efficacy for depression remains valuable.

#### 3.2.1 Neural mechanism and efficacy monitoring

Campos et al. (2024) employed fNIRS to compare immediate hemodynamic responses to tDCS stimulation of the left DLPFC between depressed patients and HCs. Analysis of pre- and post-treatment mean, minimum, and maximum values for blood oxygen saturation (SatO₂) and HBO revealed that only the depressive group exhibited increased minimum SatO₂ levels post-intervention, whilst SatO₂ and HBO remained unchanged in the control group during the intervention. This suggests an immediate effect of anodal tDCS on DLPFC hemodynamics, potentially enhancing cortical hemodynamic responsiveness. Intra- and inter-group comparisons revealed significant factorial differences only in the minimum SatO₂ ratio, further suggesting a selective effect of tDCS treatment in depressed patients, warranting further investigation into potential hypoxic or critical threshold mechanisms. The small sample size (*n* = 16) precludes definitive conclusions and warrants cautious interpretation.

In addition to conventional tDCS, a recent trial (Lu Q. et al., 2025) further compared high-definition transcranial direct current stimulation (HD-tDCS), rTMS, and standard antidepressant medication. Using fNIRS, the study measured the efficacy and cognitive modulation of HD-tDCS and rTMS in MDD patients, finding comparable overall remission rates across all three modalities. After 2-week treatment, HD-tDCS and rTMS showed greater activation in the right DLPFC and VLPFC than ADs. Consistent with these neural changes, both neuromodulation techniques also proved more effective than ADs in improving working memory, attention, executive function, and emotional regulation. HD-tDCS also produced stronger activation in the right superior temporal gyrus compared with rTMS and ADs, although this activation was not correlated with depressive symptom improvement. The authors also speculate that HD-tDCS may offer benefits in enhancing audio-visual information processing.

To further refine the stimulation targets, another study (Hernández-Sauret et al., 2025) combining HD-tDCS with fNIRS revealed the functional dissociability of the DLPFC and VLPFC in cognitive control. DLPFC stimulation produced early and sustained improvements in depressive symptoms, executive function and quality of life, whereas VLPFC stimulation yielded delayed improvements, primarily in inhibitory control. fNIRS further revealed that HD-tDCS over the DLPFC may produce delayed, spatially selective alterations in brain connectivity at the 1-month follow-up. These findings demonstrate the target specificity of prefrontal neuromodulation and highlight the utility of fNIRS for mapping region-specific regulatory effects, which aligned with earlier reports that better cognitive control and more efficient CCN activation/connectivity predict lower risk of recurrent MDD (Langenecker et al., 2018).

As a form of transcranial electrical stimulation with higher current intensity, electroconvulsive therapy (ECT) has proven to be highly effective for depression, particularly in refractory depression and in rapidly reducing suicide risk compared to other therapies (Elias et al., 2018; Gazdag and Ungvari, 2019). Nevertheless, due to the lack of understanding regarding its discharge mechanism, along with historical reports of memory impairment and hypoxaemia, clinical perspectives on ECT remain divided (De Meulenaere et al., 2018; Räsänen et al., 1988), Thus, integrating fNIRS technology with ECT to explore the treatment’s effects on cortical hemodynamics in depressed patients may help dispel misconceptions surrounding ECT.

Daniel et al. (2022) utilized dual-channel fNIRS to continuously measure regional oxygen saturation (rSO₂) in bilateral PFC during ECT. It was found that during treatment, rSO₂ sharply decreased immediately after stimulation with increased pre-oxygenation, then rebounded beyond the peak, followed by a gradual decline, but remained above baseline. Even with repeated treatments, this rSO₂ pattern was consistently observed, with measurements showing no evidence of hypoxaemia during ECT treatment. In a multivariate analysis of post-operative recovery time to orientation (PRT), the rSO₂ decrease was the sole oxygenation parameter correlated with PRT, this may serve as an objective indicator of the therapeutic seizures induced by ECT. The study demonstrates that fNIRS can monitor ECT and assist in treatment optimisation. Although informative, the sample size (*n* = 22) was modest, and replication is needed.

In a single-patient case report, Wu et al. (2024) used 53-channel fNIRS to monitor hemodynamic changes in four frontal ROIs during unilateral right ECT in a single patient. They observed progressive normalization of cortical activation, functional connectivity, and cerebral blood flow with repeated ECT sessions, along with transient lateralization changes that may relate to temporary memory impairment. The right Broca’s area showed the most prominent activation, suggesting its neuroplasticity may be associated with unilateral ECT response. As a single-case report, this study provides preliminary proof-of-concept but carries limited evidential weight relative to controlled clinical trials.

#### 3.2.2 Summary of fNIRS research on transcranial electrical stimulation

A brief overview of fNIRS studies in transcranial electrical stimulation for depression treatment is presented in (Table 3). In summary, MDD patients show impaired prefrontal hemodynamic regulation and blunted cortical oxygenation responses. tDCS appears to enhance prefrontal hemodynamic reactivity, whereas ECT normalizes cerebral oxygenation and functional connectivity in a dynamic manner. Beyond conventional tDCS, HD-tDCS demonstrates comparable efficacy to rTMS and antidepressant medications, with superior cognitive benefits and region-specific effects: DLPFC stimulation yields broader and earlier clinical improvements, whereas VLPFC stimulation primarily modulates inhibitory control. Changes in rSO₂ and SatO₂ may serve as objective markers of ECT-induced seizures, but these findings remain limited by small sample sizes, single-center designs, and lack of long-term validation, weakening their clinical generalizability. Immediate hemodynamic responses to tDCS show potential for predicting therapeutic effects, but confirmatory studies are needed. Given that ECT produces pronounced systemic hemodynamic effects, including rapid blood pressure and heart rate fluctuations, fNIRS-measured oxygenation changes may be confounded by global physiological variations. While tDCS has milder systemic effects, caution is still warranted when attributing signal changes solely to cortical neural activity. The five electrical stimulation studies reviewed (including one single-case report) had sample sizes ranging from 1 to 87 (median 26), with three studies (60%) having fewer than 30 participants. The predominance of small-sample, single-center designs without long-term follow-up warrants cautious interpretation of these findings.

**Table 3:** A brief summary of fNIRS in transcranial electrical stimulation for depression treatment.

Author	Sample	Treatment type	fNIRS instrument	Task paradigm	Brain region	Measurement index	Main conclusions
Campos et al. (2024)	16MDD, 16HCs	2 mA tDCS	Humon Hex, Dynometrics 200X	Resting state	The left supraorbital region	SatO₂ and HbO	Anodal tDCS exerted an immediate effect on DLPFC hemodynamics, suggesting enhanced cortical hemodynamic responsiveness.
Lu Q. et al. (2025)	61 patients with depression 26HCs	HD-tDCS, rTMS, and antidepressant	52-channel, ETG 4100; Hitachi	VFT	Frontotemporal cortical	HbO	HD-tDCS, rTMS and ADs have comparable efficacy, are safe and well-tolerated. HD-tDCS and rTMS are more effective for working memory, attention, executive function and emotional regulation.
Hernández-Sauret et al. (2025)	26MDD	HD-tDCS	16-channel NIRSport2, NIRx	Cognitive tasks,	DLPFC, VLPFC	HbO, HbR, brain connectivity	HD-tDCS over the DLPFC and VLPFC produces functionally dissociable effects, with DLPFC stimulation yielding earlier and broader improvements in mood, cognition, and quality of life
Daniel et al. (2022)	22 patients with depressive syndromes	ECT	2-channel INVOS cerebral/somatic oxymeter	Resting state	Prefrontal cortex	Regional oxygen saturation (rSO2)	The rSO₂ decrease may serve as an objective indicator of therapeutic seizures induced by ECT.
Wu et al. (2024) Case report (*n* = 1)	One MDD male patient	ECT	53 multi-channel, BS-3000		FPA, The frontal eye field (FEF), DLPFC, BROCA	Task beta, integral, and centroid values	fNIRS metrics revealed that ECT significantly alters cerebral hemodynamics and functional connectivity in this patient.

## 4 The application of fNIRS in psychological intervention for depression treatment

Cognitive behavioral therapy (CBT) is an effective mental health intervention widely employed in treating conditions such as depression, anxiety, and post-traumatic stress disorder. Through short-term, structured treatment, it assists individuals in modifying maladaptive thought and behavioral patterns, thereby regulating emotions and actions. Research indicates that integrating mindfulness-based elements into classical CBT demonstrates therapeutic efficacy for major depressive disorder (Clarke et al., 2015). fNIRS studies on psychological intervention for depression are also gradually being carried out.

### 4.1 Neural mechanism and efficacy monitoring

Laicher et al. (2023) investigated the efficacy and neural mechanisms of mindfulness-based emotion regulation training (MBERT) based on fNIRS technology on depressive rumination in 42 patients with MDD were explored. Following an 8-session MBERT program, patients demonstrated improvements in depression severity (BDI-II), General Self-Efficacy Scale (GSE), Self-Compassion Scale (SCS), and Self-Reflection Questionnaire (SRQ) scores. Throughout the emotion regulation treatment, cortical activation levels in the bilateral inferior frontal gyrus (IFG) and DLPFC remained consistently higher than in the control group. Concurrently, cortical oxygenation levels in the bilateral DLPFC gradually decreased in both the experimental and control groups. These brain regions are associated with the cognitive control network (CCN) responsible for regulating attention and problem-solving (Rosenbaum et al., 2016). The findings indicate that CBT-based emotion regulation programs activate prefrontal regions involved in the CCN during the initial treatment phase. As therapy progresses, CCN activation diminishes, accompanied by a reduction in patients’ subjective sense of effort and burden, alongside gradual increases in self-compassion and inner peace. This also suggests that future research could combine neurostimulation techniques targeting relevant brain regions with emotion regulation strategies to accelerate treatment onset.

Unlike MBERT, which focuses on cognitive reappraisal, a study (Haipt et al., 2024) comparing hypnotherapy (HT) with cognitive behavioral therapy (CBT) for depression using fNIRS found that both interventions significantly reduced depressive symptoms. On a neurophysiological level, exploratory analyses revealed two tentative effects within the cortical DMN: (1) FC within the somatosensory association cortex (SAC) decreased over time regardless of therapy group, and (2) FC between the right angular gyrus and left supramarginal gyrus increased specifically in the HT group. However, neither effect survived correction for multiple testing, indicating that these neural differences are not statistically robust. The authors conclude that while CBT and HT may engage different neural mechanisms, and further hypothesis-driven research is needed to validate these findings.

Unlike CBT, which focuses on correcting maladaptive cognitions and improving negative emotional behaviors, solution-focused brief therapy (SFBT) shifts attention to the client’s future vision, strengths, and resources. By examining both the problem and the client’s favorable conditions, it helps uncover positive aspects and develop action plans to achieve goals (Franklin and Hai, 2021). Chen et al. (2024) evaluated the effects of ten SFBT sessions on executive function and therapeutic efficacy in adolescents with major depressive disorder (MDD). Primary outcome measures included patients’ PHQ-9 and GAD-7 scores, alongside mean HBO activity values from fNIRS during VFT. Results exhibited that following the third intervention session, the SFBT group showed significant reductions in PHQ-9 and GAD-7 scores alongside markedly improved VFT performance, with sustained improvement observed after the sixth and tenth sessions. Compared to the standard psychotherapy group, the SFBT cohort showed faster PHQ-9 score reduction, superior VFT task performance, and a more pronounced gradual increase in mean active HBO values within the DLPFC region following the tenth treatment session. The findings indicate that compared to traditional psychodynamic therapy, SFBT proves more effective in the short term for ameliorating emotional and cognitive symptoms in adolescents with depression, particularly in relation to changes in the DLPFC region.

In another study examining post-stroke depression (PSD) patients, Nguyen et al. (2025) addressed the issue of poor adherence to antidepressant medication among PSD patients (Krivoy et al., 2017), utilized fNIRS technology to monitor the efficacy of a three-month motivational interviewing (MI) program combined with home-based rehabilitation interventions for PSD patients. Analysis revealed that in comparison with the control group receiving standard treatment, the intervention group demonstrated more significant increases in HbO concentration in the left OFC and its surrounding regions, alongside more pronounced reductions in PHQ-9 scores. This further confirms that functional improvement in the OFC region correlates with the alleviation of depressive symptoms (Xie et al., 2021), as a person-centered intervention, MI enhances patients’ intrinsic motivation to resolve problems (Hettema et al., 2005), demonstrating considerable therapeutic value for emotional difficulties in stroke patients.

### 4.2 Summary of fNIRS research on psychological interventions

A brief overview of fNIRS studies in psychological interventions for depression treatment is presented in (Table 4). In summary, MDD patients display weakened DLPFC and IFG activation and disrupted cognitive control network connectivity. Mindfulness-based training (MBERT) initially activates this network, with activation decreasing as therapy progresses—suggesting reduced cognitive effort. Solution-focused brief therapy (SFBT) enhances DLPFC-related executive function, and motivational interviewing improves OFC function. An emerging comparison between HT and CBT suggests that these therapies may engage partially distinct neural substrates, with tentative fNIRS evidence pointing to differential modulation of the cortical default mode network, although these effects require replication due to multiple-testing concerns. Increased prefrontal HbO and task-related activation reflect emotional and cognitive improvement. The four psychological intervention studies had a median sample size of 77.5 (range 53–84), all exceeding 30 participants. Despite adequate sample sizes, heterogeneity in therapy protocols and the absence of validated fNIRS-based predictive biomarkers remain notable limitations.

**Table 4:** A brief summary of fNIRS in psychological intervention for depression treatment.

Author	Sample	Treatment type	fNIRS instrument	Task paradigm	Brain region	Measurement index	Main conclusions
Laicher et al. (2023)	42MDD, 42HCs	Mindfulness-based Emotion Regulation Training (MBERT).	A continuous wave, multichannel, Hitachi	During all therapeutic training sessions	IFG, DLPFC, somatosensory association cortex (SAC)	HbO, HbR	The bilateral IFG and DLPFC showed consistently greater activation during emotion regulation training relative to control trials.
Haipt et al. (2024)	75 depressed patients	Hypnotherapy, CBT	52-channel, ETG-4000, Hitachi	Resting-state	Parietal and temporal brain areas	FC in DMN	The neural mechanisms underlying hypnotherapy and CBT in depression may involve distinct targets within the default mode network.
Chen et al. (2024)	53adolescents diagnosed with MDD	Solution-Focused Brief Therapy (SFBT)	Multi-channel, NirScan, Danyang Huichuang	VFT	Bilateral prefrontal cortex.	HbO	SFBT effectively ameliorates emotional and cognitive symptoms in depressed adolescents, associated with DLPFC changes
Nguyen et al. (2025)	80post-stroke depression	Motivational Interviewing (MI) and Home Rehabilitation	48-channel, NIRSIT	VFT	DLPFC, VLPFC, FPC, OFC	HbO	Depressive symptom alleviation is linked to functional improvement in the OFC.

## 5 The application of fNIRS in integrated interventions for depression treatment

### 5.1 Neural mechanism and efficacy monitoring

Meta-analyses indicate a significant positive correlation between executive function deficits and the severity of depression (McDermott and Ebmeier, 2009), Numerous neuroimaging and neuropsychological studies have localized executive function to the frontal lobe, particularly the PFC (Berla and Koeppen, 2014). Exercise as an alternative therapy for mild to moderate depression (Parker and Crawford, 2007), combined with cognitive training, demonstrates certain improvements in brain function (Zhu et al., 2016).

Crum et al. (2022) utilized fNIRS to evaluate exercise’s impact on the PFC among individuals with depressive symptoms, and whether physical fitness level influences PFC responsiveness to exercise. Including 106 healthy adults, the study administered the Mood and Feeling Questionnaire (MFQ), cardiopulmonary exercise testing (CPET), neurocognitive tests, and fNIRS to assess depressive symptoms, aerobic fitness, executive function, and PFC hemodynamic changes. Results revealed exercise-induced activation changes in the bilateral DLPFC (BA46/9) and ventrolateral PFC (BA44/45), with the right rostral PFC (BA10) showing the largest changes. HBO changes in the right inferior frontal gyrus (IFG), a ventrolateral PFC subregion, were negatively correlated with depression scores. Higher aerobic fitness (VO₂ peak) was associated with greater post-exercise activation changes in the right rostral PFC (BA10), indicating that fewer depressive symptoms are significantly associated with reduced responsiveness of the right IFG to exercise. This region may therefore be key to predicting the antidepressant effects of exercise, while the rostral PFC may play a critical role in both the pathophysiology of depression and its recovery. Finally, the authors speculated that due to compensatory or regulatory functions within the PFC, physically healthy individuals may still experience depression, suggesting no clear association between physical fitness and depressive symptoms.

With increasing exploration of non-pharmacological interventions, nature-based therapies including forest and horticultural therapy have gained prominence. These approaches demonstrate positive effects on stress reduction, emotional regulation, alleviation of depressive and anxiety symptoms, and enhancement of cognitive function in healthy individuals (Rosa et al., 2021). Mizumoto et al. (2024) employed dual-channel fNIRS targeting the OFC region, concurrently monitoring heart rate variability. Focusing on the readily accessible intervention of “virtual nature visual stimulation,” they investigated its effects on emotional improvement in patients with anxiety and depression, along with the associated neural and physiological mechanisms. Patients in the depression group exhibited a significant increase in OFC HbO₂ concentration following viewing nature images, corresponding to improvements in BDI scale scores, and this effect was independent of depression severity. Conversely, in the anxiety group, the reduction in HbO₂ correlated with the degree of emotional improvement. It is suggested that fNIRS can effectively quantify the antidepressant-related neural effects of virtual natural stimuli, with OFC activation or inhibition being one of the underlying aetiologies of anxiety and depression.

Beyond virtual nature stimuli, real-world nature-based interventions such as therapeutic gardens have also been shown to modulate prefrontal hemodynamics in depressed individuals. Olszewska-Guizzo et al. (2022) employed a multimodal EEG-fNIRS setup to examine prefrontal and occipital hemodynamic responses in depressed adults and healthy controls during 1-min passive exposure to a therapeutic garden (TG) with contemplative design features. Data indicated that, in the TG, both participant groups exhibited marginally reduced HbO levels correlated with the observed mood improvement in the prefrontal cortex, along with positive alterations in EEG patterns. The researchers speculate that the restorative effects (Lee, 2017) and decreased rumination (Souter-Brown et al., 2021) may be linked to the observed mood improvement.

In another novel non-pharmacological intervention study, Lu and colleagues (Lu X. et al., 2025) quantified the effects of virtual reality (VR) cognitive training on working memory (WM) and frontotemporal function in MDD patients using fNIRS. Results showed that compared to the control group, the intervention group exhibited a more significant reduction in HAMD scores post-training, reflected in higher scores on the Digit Span Test (DST), a measure of cognitive function. Significant group-by-time interactions were observed in fNIRS-derived local efficiency and clustering coefficient measurements of the frontotemporal region. Moreover, improvements in DST total scores positively correlated with changes in clustering coefficient. Given prior research indicating a positive correlation between WM impairment severity and depression severity (Duque and Vázquez, 2015), This study posits that VR-based cognitive training enhances functional connectivity within the frontal-temporal lobe of patients with depression, thereby improving working memory (WM) performance and facilitating recovery. The observed increase in frontal-temporal lobe clustering coefficients may represent one potential neural mechanism and indicator for assessing how VR-WM training enhances WM breadth in depressed individuals. Notably, the time-group effect for the Massachusetts General Hospital Cognitive and Physical Function Questionnaire (MGH-CPFQ), which measures cognitive impairment severity, was non-significant. This underscores the need for greater emphasis on objective assessment tools, including fNIRS.

### 5.2 Summary of fNIRS research on integrated interventions

A brief overview of fNIRS studies in integrated interventions for depression treatment is presented in (Table 5). In summary, MDD patients show reduced prefrontal reactivity to external interventions and abnormal frontotemporal network efficiency. Exercise modulates PFC hemodynamic responsiveness, with blunted HbO responses in the right IFG associated with more severe depressive symptoms. Virtual nature visual stimulation activates the OFC, and real-world therapeutic garden exposure, as assessed by multimodal EEG-fNIRS, corroborates these mood-improving effects with prefrontal hemodynamic changes. VR-based cognitive training enhances frontotemporal clustering coefficients—a potential mechanism for working memory improvement. These integrated interventions are promising, but most studies are preliminary (sample sizes 30–106, largely healthy or mixed samples), and none have established validated predictive biomarkers. Exercise inherently elicits robust systemic hemodynamic responses (e.g., increased heart rate, blood pressure, and cerebral blood flow), which can substantially influence fNIRS signals independent of cortical neural activation. Consequently, HbO changes observed in exercise-based interventions should not be directly equated with localized brain activation without accounting for peripheral physiological confounds. The three integrated intervention studies with extractable sample sizes had a median of 60 (range 57–92), one additional study (Crum et al.) did not report separate group. However, several studies included healthy or mixed samples rather than clinically diagnosed depression populations, limiting the generalizability of findings to clinical settings.

**Table 5:** A brief summary of fNIRS in integrated interventions for depression treatment.

Author	Sample	Treatment type	fNIRS instrument	Task paradigm	Brain region	Measurement index	Main conclusions
Crum et al. (2022)	Depressed mood assessed continuously, no group sizes.	Cardiopulmonary exercise test (CPET)	22-channel, LIGHTNIRS, Shimadzu Corp.	Neurocognitive testing	Rostral PFC, DLPFC, VLPFC.	HBO and HbR HBT	Individuals with more severe depressive symptoms exhibit reduced prefrontal cortex hemodynamic responses to acute exercise.
Mizumoto et al. (2024)	30 each with depressive disorder and 30 anxiety disorder	Visual stimuli of natural environments	PocketNIRS DUO (2ch) from Dynasense	Visual stimulation task	OFC	HbO	fNIRS quantifies neural responses to virtual natural stimuli, revealing that OFC activation or inhibition may contribute to the etiology of anxiety and depression.
Olszewska-Guizzo et al. (2022)	24 clinically depressed, 68 HCs	Passive exposure to three urban spaces	48-channel, two portable NIRS SPORT, NIRx	Watch the landscape	Frontal and occipital area	HbO	Passive exposure to therapeutic gardens can improve mood in both individuals with depression and healthy populations
Lu X. et al. (2025)	57 patients with MDD	Virtual reality-based working memory (VR-WM) training	48-channel, NirSmart, Danyang Huichuang	Resting state	Bilateral frontotemporal lobes	fNIRS-derived local efficiency and clustering coefficient	VR-WM training effectively enhanced inhibition, updating, and memory span in MDD, an improvement paralleled by increased frontotemporal functional connectivity.

## 6 Application of fNIRS in assessing antidepressant efficacy within the field of integrated Chinese and Western medicine

### 6.1 Neural mechanism and efficacy monitoring

Research and analysis have reported that integrating Western symptomatic treatment with the syndrome differentiation and holistic principles of integrated Chinese and Western medicine yields superior intervention outcomes for alleviating depressive symptoms (Zhao et al., 2024; Kou and Chen, 2012). Separate analyses indicate that compared to the use of Western medicine alone, treatments grounded in traditional Chinese medicine can effectively reduce the severity of post-stroke depression whilst exhibiting fewer adverse effects (Lam Ching et al., 2023). Quantifying the antidepressant effects of traditional Chinese medicine therapies through non-invasive brain functional imaging represents a novel field of research. Wei et al. (2023) employed fNIRS equipment to explore the alleviating effects of Cang Ai volatile oil (CAVO) on mild to moderate depression and its neural mechanisms. Previous animal studies indicated this herbal formulation influences central metabolism of dopamine (DA) and 5-hydroxytryptamine (5-HT)(Chen et al., 2019) and exhibits antidepressant effects (Zhang K. et al., 2021). Research found that following CAVO intervention, patients exhibited a significant increase in HbO₂ concentration within the right frontal pole cortex, a brain region closely associated with emotional processing and executive function (Bramson et al., 2020), Moreover, the magnitude of HbO₂ concentration change showed a significant positive correlation with improvements in HAMD scores, suggesting a direct association between alleviated depressive symptoms and heightened excitability in the right frontal pole cortex. The fNIRS data thus provides objective neurobiological corroboration for the efficacy of this inhaled Chinese herbal compound formulation.

As a vital therapy within traditional Chinese medicine, acupuncture has gained widespread acceptance in modern clinical practice and is recognized as a safe treatment with adjunctive therapeutic benefits (Chan et al., 2015). Its advanced form, electroacupuncture (EA), employs specialized equipment to deliver pulsed electrical currents, synchronously stimulating the neuromuscular system to integrate the dual effects of traditional acupuncture and electrophysiological therapy (Yang L. et al., 2022). Yuan et al. (2025) utilized fNIRS equipment to compare the efficacy of pharmacotherapy alone versus pharmacotherapy combined with standardized EA treatment for depression, employing acupoints including Baihui, Yintang, and Sishencong. Post-intervention assessment revealed that the combined therapy group had significantly lower PDQ-D and HAMD scores, as well as higher cognitive task accuracy rates, relative to the control group. Furthermore, significant time × group interactions were observed for all outcome measures. Regarding neural correlates, fNIRS data indicated that the combined therapy group showed significant post-intervention increases in HBO concentration, integral values, and centroid values compared to their own baseline. These changes were supported by significant main effects of time and time × group interactions. Given the significant symptom reduction in the combined therapy group and the neuroanatomical relevance of the acupoints, the authors hypothesize that EA modulates top-down regulation within the frontal-limbic circuitry. This action is posited to enhance hippocampal neuroplasticity, which ultimately leads to the improvement of emotional and cognitive deficits (Wang Y. et al., 2025). Complementing electroacupuncture findings, a resting-state fNIRS study (Wong et al., 2021) comparing antidepressant monotherapy with acupuncture-augmented combination therapy demonstrated superior clinical improvement in the combination group. At the neural level, combined treatment was associated with more extensive normalization of frontotemporal functional connectivity, particularly involving the DLPFC. These results indicate that acupuncture may enhance the neuroregulatory effects of pharmacotherapy by modulating interactions between the default mode network and cognitive control networks, and that resting-state fNIRS can capture the additive neural benefits of integrated Chinese–Western treatment regimens. In addition to combination therapy, acupuncture alone also significantly increases prefrontal HbO concentration in MDD patients (Zhang T. et al., 2021). Its activation pattern partially overlaps with that of Western pharmacotherapy, yet its therapeutic effect is more oriented toward an immediate frontal excitatory response, and this effect is more pronounced in patients with severe depression.

Contrary to the findings of the two aforementioned research discussions regarding the antidepressant effects of Chinese medicine, Chen G.-F. et al. (2025) focused on syndrome differentiation and treatment for depression. They pioneered an investigation into fNIRS characteristics during the VFT task and underlying neural mechanisms in patients with depression diagnosed as Liver Qi Stagnation syndrome before and after treatment. Results indicated that HAMD scores decreased marginally post-treatment compared to pre-treatment, yet showed significant reduction at follow-up assessment. Hemodynamic assessments before and after treatment revealed statistically significant signal differences in bilateral DLPFC and medial prefrontal cortex (mPFC) recordings. At the three-month follow-up, improved activation in the left DLPFC persisted, indicating that activation in this brain region improves with treatment and can be maintained stably. This study utilized fNIRS to translate the abstract TCM syndrome pattern of “Liver Qi Stagnation” into quantifiable brain functional indicators. It validated that frontal hemodynamic activity may serve as a potential biomarker reflecting changes in TCM syndromes before and after depression treatment, thereby providing a preliminary yet significant paradigm for modernizing and objectifying research into TCM affective disorders.

### 6.2 Summary of fNIRS research on integrated Chinese and Western medicine

A brief overview of fNIRS studies assessing antidepressant efficacy within integrated Chinese and Western medicine is presented in (Table 6). In summary, MDD patients exhibit reduced cortical excitability in the frontal pole and DLPFC. Cang-Ai volatile oil increases right frontal pole HbO, correlating with HAMD improvement. Acupuncture, both as an add-on to pharmacotherapy and as a monotherapy, enhances prefrontal activation and functional connectivity, with resting-state fNIRS further revealing more extensive normalization of frontotemporal network interactions when combined with SSRIs. Electroacupuncture combined with pharmacotherapy enhances prefrontal activation and connectivity, possibly via frontal-limbic circuit modulation. Traditional Chinese medicine syndrome differentiation (e.g., Liver Qi Stagnation) can be quantified with fNIRS, showing sustained left DLPFC activation after treatment. The five integrated Chinese and Western medicine studies had a median sample size of 47 (range 35–120), all exceeding 30 participants. Although these findings suggest that fNIRS may help objectify TCM diagnoses, predictive biomarkers require validation in larger, prospective studies.

**Table 6:** A brief summary of fNIRS in assessing antidepressant efficacy within the field of integrated Chinese and Western medicine.

Author	Sample	Treatment type	fNIRS instrument	Task paradigm	Brain region	Measurement index	Main conclusions
Wei et al. (2023)	46 subjects mild to moderate depression	Cang-Ai Volatile Oil (CAVO)	48 channels, NirSmart-3000DS, Hui Chuang	VFT	The prefrontal and temporal cortex	HbO	Inhaling CAVO significantly alleviates depressed mood and enhances cortical excitability, especially in the right frontal pole—a region closely linked to emotional processing and executive function.
Yuan et al. (2025)	120MDD	Pharmacotherapy combined with electroacupuncture (EA)	48-hannel NirSmart-3000DS, Huichuang	Cognitive task	The prefrontal cortex	HbO, integral values, and centroid values.	Pharmacotherapy with electroacupuncture enhanced short-term clinical outcomes and cortical activation in depression.
Wong et al. (2021)	70Depression	Pharmacotherapy combined with acupuncture	18 channels, NIRSport, NIRx Medical, USA	Resting state	DLPFC	HbO, HbR, and HbT, rsFC	Compared with antidepressants alone, adjunctive acupuncture produced greater reductions in depressive symptoms and significantly enhanced rsFC in the DLPFC
Zhang T. et al. (2021)	47MDD	Acupuncture	52-channel, Hitachi Medical Co., Japan	VFT	DLPFC, frontopolar area	HbO	single acupuncture session showed a trend toward increased frontal pole activation in severe MDD patients, symptom severity was significantly associated with left dlPFC activation
Chen G.-F. et al. (2025)	35liver qi stagnation	SSRIs	45-channel, FOIRE3000	VFT	Bilateral prefrontal cortex	HbO	fNIRS-based physiological metrics may provide a more objective and sensitive assessment of improvement in liver-qi stagnation depression than traditional ethological instruments

## 7 Summary and outlook

This review systematically collates 34 publications to comprehensively elucidate the application of near-infrared functional brain imaging in evaluating the antidepressant efficacy of pharmacological therapies, physical therapies, psychological therapies, integrated interventions, and combined traditional Chinese and Western medical treatments. Overall, existing research strongly demonstrates that fNIRS, as a low-cost, non-invasive, portable, and ecologically valid brain imaging technique, can accurately capture the specific effects of different interventions on brain function by monitoring changes in HBO concentration and functional connectivity within brain networks. It provides detailed information on neural activity (Johnson et al., 2012). It is not only suitable for outpatient follow-ups, long-term dynamic monitoring, and special populations (such as patients unable to tolerate MRI), but also demonstrates irreplaceable advantages in designing real-time brain network regulation assessments in active behavioral and natural environments (such as exercise, VR, and cognitive training). fNIRS technology provides valuable visual information for evaluating patient treatment responses. By objectively measuring treatment progress and providing rational explanations, patients are more likely to maintain treatment motivation. Clinicians may utilize fNIRS data to adjust subsequent intervention protocols. For patients exhibiting slower responses or suboptimal outcomes, timely modifications to medication dosages, physiotherapy parameters, or psychological intervention strategies can be implemented. This facilitates the development of more personalized treatment plans, potentially enhancing therapeutic efficacy and enabling more rational allocation of healthcare resources (Abo Aoun et al., 2023).

Across diverse treatment modalities, specific functional subregions of the prefrontal and temporal cortices are frequently selected as regions of interest (ROIs). Changes in hemodynamic and functional connectivity metrics within these ROIs from pre- to post-treatment often correlate closely with improvements in clinical scores and depressive symptoms. Specifically, effective treatment—irrespective of the therapeutic approach—is commonly associated with the restoration or enhancement of cerebral blood flow signals and the normalization of functional connectivity in these regions. These observed hemodynamic alterations suggest underlying reductions in neuronal activity and impaired neurovascular coupling, which may constitute the neural basis for the cognitive deficits and emotional dysregulation observed in depression (Zhang F. et al., 2023). Consistent with previous research, the prefrontal cortex is crucial for executive function, decision-making and emotional regulation; reduced blood flow and oxygenation in this region are closely associated with depressive symptoms (Veeraiah et al., 2014; Friedman and Robbins, 2022). Individuals with depression exhibit reduced bilateral frontotemporal activation, with those displaying pronounced depressive symptoms showing marked decreases in left frontotemporal activation (Akiyama et al., 2018). Impaired monoaminergic fiber tracts connecting the prefrontal cortex with other brain regions, leading to deficits in functional network connectivity, is also considered one of the etiological factors of depression (Krick et al., 2023). Alterations in FC between brain networks are increasingly associated with antidepressant treatment outcomes (Broeders et al., 2024; Sun et al., 2024). Kang et al. (2026) utilized fMRI to investigate resting-state functional connectivity (RSFC) patterns associated with antidepressant treatment outcomes. They found significantly increased connectivity between the posterior cingulate cortex (PCC) and subgenual anterior cingulate cortex (sgACC) in responders compared to non-responders, particularly involving the prefrontal cortex, thalamus, amygdala, and PCC exhibiting markedly higher FC. Moreover, prior study (Duan et al., 2012) computational analyses revealed that bilateral sensorimotor region RSFC intensity derived from fNIRS, alongside brain network metrics derived from graph theory, aligned with intensity measured via fMRI. This suggests that integrating resting-state fNIRS with graph theory for investigating cerebral FC is both feasible and effective.

A review of the aforementioned studies suggests that the neural mechanisms underlying different treatment modalities may exhibit both similarities and differences. Research on antidepressant medication treatment has revealed reduced activation levels in multiple prefrontal brain regions post-treatment compared to baseline. Conversely, literature on rTMS treatment indicates enhanced frontal lobe activation following therapy, although both approaches are associated with clinical symptom improvement. Unlike antidepressants, which indirectly modulate brain function by increasing the bioavailability of monoamine neurotransmitters (such as serotonin and noradrenaline) in the synaptic cleft, non-invasive physical therapies like rTMS directly influence neuronal activity by applying external magnetic fields to neural circuits (Rosa et al., 2006), The two approaches exert differing speeds of influence on neural plasticity, with rTMS demonstrating a more rapid activation effect on target brain regions. Consequently, the divergence in reported outcomes is understandable, precisely illustrating the distinct mechanisms by which different interventions improve brain function in patients with depression. This suggests that future research may combine multiple therapeutic approaches to achieve synergistic and complementary regulation of neural pathways within target brain regions.

In the studies discussed, over half of the researchers assessed brain activity through changes in blood oxygenation signals during the VFT, demonstrating that the fNIRS-VFT paradigm serves as a low-cost, easily operable tool for investigating functional regions including the PFC in patients with psychiatric disorders (Yeung and Lin, 2021). Yang J. et al. (2022) employed four tasks to investigate hemodynamic characteristics in MDD patients during motor, affective, cognitive, and combined tasks. They found that differences in hemodynamic features between MDD patients and healthy controls were closely related to task selection, with combined tasks demonstrating superior discriminative power compared to single tasks. Future research may employ additional paradigms to enrich task-state fNIRS data, thereby more comprehensively elucidating cerebral oxygenation changes.

Several limitations exist in the discussed studies. Firstly, sample sizes varied considerably across included studies for which sample sizes could be extracted (median 54, range 1–121). While studies in pharmacological, psychological, integrated, and TCM-integrated interventions generally employed larger samples (median ≥ 47 per category), 6 studies (18%) had sample sizes under 30, with the majority of these small-sample studies (5 of 6) concentrated in the physical therapy domain (rTMS/iTBS and tDCS/ECT). In addition, several studies lacked control groups and one was a single-case report (Wu et al., 2024). These design weaknesses limit the generalizability of findings, particularly for non-invasive brain stimulation modalities, and highlight the need for larger, well-controlled trials to confirm the observed effects. Secondly, publication bias should be considered in the present review. Studies demonstrating significant fNIRS-related changes are more likely to be published, whereas null or negative findings tend to be underreported. Thirdly, while changes in HbO are widely interpreted as cortical activation, the relationship between fNIRS hemodynamics and underlying neural activity is complex. Systemic physiological confounds, including blood pressure fluctuations, respiratory variation, and autonomic arousal, may contribute to signal changes that mimic cortical activation. This is particularly relevant for interventions such as exercise, ECT, and rTMS, which induce prominent systemic hemodynamic effects. HbO changes should therefore be interpreted as indirect markers of neural modulation rather than direct measures of neuronal activity. Moreover, variations existed across studies in fNIRS equipment brands, generations, channel counts, brain region localisation, and task paradigms (verbal fluency, n-back, resting state), with no standardized testing protocols, hindering direct cross-study comparisons. Most studies focus on descriptive analyses of brain region activation changes, with limited exploration of “associations between brain functional alterations and neurotransmitters, neurotrophic factors (BDNF), gut microbiota, etc.” Finally, although machine learning represents a promising direction for fNIRS research, current applications (e.g., Cyrus et al.) remain superficial. Key challenges including overfitting in small samples, appropriate cross-validation strategies, and the gap between research classification accuracy and real-world clinical utility have not been fully addressed. Presenting machine learning as a “key breakthrough” without acknowledging these fundamental constraints is premature. Future studies will require rigorous validation, larger multi-center datasets, and clinically meaningful endpoints to improve translational value.

Although fNIRS holds promise for identifying neurobiological markers and predicting antidepressant response, with the rapid development of artificial intelligence (AI) in precision psychiatry, further research is needed to translate these findings into tools for clinical application. Recent advances in interpretable artificial intelligence (XAI) in clinical decision support systems (CDSS), as discussed in a related meta-analysis (Abbas et al., 2025) highlight a potential challenge: Machine-learning models that predict treatment response on the basis of fNIRS features, such as the model of Cyrus et al., can be “black boxes” with opaque internal decision processes that limit model transparency and clinical trust. To be used in daily clinical practice, the model must be interpretable. In other words, without a clear description of which cerebral hemodynamic features influence predictions, such models are difficult to reliably integrate into clinical decision making. Therefore, future fNIRS studies should combine predictive modeling with XAI techniques to clarify the neurobiological basis of model outputs.

In parallel, the broader landscape of AI applications in mental health provides a clear roadmap for the future development of fNIRS research. A comprehensive narrative review (Ali et al., 2025) emphasized that the field is rapidly shifting toward multimodal data fusion, integrating fNIRS with EEG, fMRI, clinical scales, wearable sensors, and digital phenotyping to improve the robustness and generalisability of predictive models. This reinforces the need to move beyond single-modality fNIRS analysis toward comprehensive AI frameworks that support objective diagnosis, real-time treatment monitoring, and individualized response prediction. Key priorities include establishing large-scale multi-center fNIRS datasets, developing adaptive AI systems for personalized neuromodulation, and addressing ethical, privacy, and regulatory challenges. By aligning fNIRS biomarker research with these emerging trends in mental health AI, the field can accelerate the translation of fNIRS from a research tool into a clinically deployable technology for precision psychiatry.

## Glossary

ACC: Anterior Cingulate Cortex
AI: Artificial Intelligence
CBT: Cognitive Behavioral Therapy
CCN: Cognitive Control Network
CEN: Central Executive Network
DLPFC: Dorsolateral Prefrontal Cortex
EA: Electroacupuncture
ECT: Electroconvulsive Therapy
FC: Functional Connectivity
FDA: Food and Drug Administration
fNIRS: Functional Near-Infrared Spectroscopy
FPPFC: Frontal Polar Prefrontal Cortex
HbO: Oxyhemoglobin
HbR: Deoxyhemoglobin
HAMD: Hamilton Depression Rating Scale
HD-tDCS: High-definition transcranial direct current stimulation
IFG: Inferior Frontal Gyrus
iTBS: intermittent Theta Burst Stimulation
LI: Laterality Index
MADRS: Montgomery-Asberg Depression Rating Scale
MBERT: Mindfulness-Based Emotion Regulation Training
MDD: Major Depressive Disorder
OFC: Orbitofrontal Cortex
PCC: Posterior Cingulate Cortex
PD-D: Parkinson’s disease comorbid with depression
PFC: Prefrontal Cortex
PPC: Posterior Parietal Cortex
PSD: Post-Stroke Depression
rTMS: Repetitive Transcranial Magnetic Stimulation
SFBT: Solution-Focused Brief Therapy
SN: Salience Network
SSRI: Selective Serotonin Reuptake Inhibitor
tDCS: transcranial Direct Current Stimulation
TCM: Traditional Chinese Medicine
VFT: Verbal Fluency Task
VR: Virtual Reality
WM: Working Memory
XAI: Explainable Artificial Intelligence
